# MicroRNA-146a Alleviates Experimental Autoimmune Anterior Uveitis in the Eyes of Lewis Rats

**DOI:** 10.1155/2017/9601349

**Published:** 2017-12-24

**Authors:** Yung-Ray Hsu, Shu-Wen Chang, Yu-Cheng Lin, Chang-Hao Yang

**Affiliations:** ^1^Department of Ophthalmology, Far Eastern Memorial Hospital, No. 21, Sec. 2, Nanya South Road, Banqiao, New Taipei City 22056, Taiwan; ^2^Department of Ophthalmology, National Taiwan University Hospital, No. 7, Chung-Shan South Road, Taipei 10002, Taiwan; ^3^College of Medicine, National Taiwan University, No. 7, Chung-Shan South Road, Taipei 10002, Taiwan

## Abstract

**Purpose:**

This study aimed to determine the effect and roles of microRNA (miRNA, miR) treatment in experimental autoimmune anterior uveitis (EAAU).

**Materials and Methods:**

Uveitis was induced in Lewis rats by simultaneous injections of bovine melanin-associated antigen into the hind footpad and the intraperitoneal cavity. The animals were injected intravitreally with low-dose (0.5 *μ*g) or high-dose (1.5 *μ*g) miR-146a. The clinical scores, leukocyte count in the aqueous humor, and histology were assessed. Cytokine changes were evaluated by relative mRNA expression and enzyme-linked immunosorbent assay (ELISA). The expression of nuclear factor kappa B (NF-*κ*B) was assessed by immunofluorescence and Western blotting. Evaluation of the DNA-binding activity of NF-*κ*B was performed by electrophoretic mobility shift assay (EMSA).

**Results:**

Treatment with miR-146a significantly attenuated clinical scores and leukocyte infiltration in a dose-dependent manner, a result that was compatible with histological findings. Following miR-146a injections, downregulation of interleukin- (IL-) 1*β*, IL-6, and IL-12 and interferon- (IFN-) *γ* and upregulation of IL-10 and IL-17 were noted. The decreased NF-*κ*B expression on immunofluorescence and Western blotting and reduced DNA-binding activity on EMSA were demonstrated following miR-146a treatment.

**Conclusions:**

miR-146a effectively reduced intraocular inflammation in EAAU through the inhibition of NF-*κ*B. miR-146a might be a new treatment choice for uveitis.

## 1. Introduction

Uveitis broadly refers to intraocular inflammation. Anatomically, 43% to 70% of these cases are anterior uveitis [1, 2, 3]. Currently, corticosteroid treatment is the mainstay. However, this strategy is cumbersome in that recurrent disease attacks and prolonged steroid use often lead to ocular complications and deterioration of vision [4, 5]. Systemic immunomodulatory agents with steroid-sparing effects are therefore needed in selected cases. Nevertheless, hepatic, renal, or bone marrow adverse events are not uncommon [6, 7]. Therefore, new methods of local treatment with steroid-sparing effects are highly desired.

Because of the complexity and heterogeneity of the pathogenesis of uveitis, some animal models have been developed. Experimental autoimmune anterior uveitis (EAAU) in Lewis rats is characterized by exclusively anterior segment inflammation without chorioretinal involvement [8]. EAAU often runs a predictable clinical course, with disease onset on day 11 postimmunization, peak inflammation on days 15 to 19, and recovery on day 30 [9]. Therefore, EAAU is an ideal model for a study of human acute anterior uveitis (AAU). In the clinical practice, human AAU subjects suffer from multiple fulminant recurrent attacks rather than chronic disease. Meanwhile, the exact trigger of each recurrence is often elusive. Therefore, local nonsteroid immunotherapy, which prevents disease attack, would be of high clinical value.

Immunologically, activation of nuclear factor kappa B (NF-*κ*B) with further participation of innate and adaptive immunity, especially Th1 and Th17 lineages, is considered crucial in various models of uveitis, including EAAU [10–14]. NF-*κ*B can be regulated by numerous molecules. Among them, microRNAs (miRNAs, miRs) have recently attracted attention. miRNAs are small, noncoding RNA molecules that function as posttranscriptional regulators [15]. Some miRNAs are deeply involved in immunity through NF-*κ*B pathways [16, 17]. Specifically, miR-146a is regarded as a key immunological player. By attenuating tumor necrosis factor (TNF) receptor-associated factor 6 (TRAF6) and interleukin (IL)-1 receptor-associated kinase 1 (IRAK1), miR-146a dampens downstream NF-*κ*B expression and finally inhibits inflammation [18, 19]. Dynamic changes of miRNA expression have been noted in some uveitis models [20, 21]. In our previous work, the expression of miR-146a was significantly downregulated in the course of EAAU. Meanwhile, the downregulation profile of miR-146a was noted much earlier than the cytokine changes and clinical inflammation [11]. We proposed that loss of miR-146a inhibition leads to overexpression of NF-*κ*B, resulting in the disease. In contrast to our results, the expression of miR-146a was significantly elevated in experimental autoimmune uveoretinitis (EAU) in one study [22]. The author proposed that following the emergence of uveitis, the expression of miR-146a may act as a negative regulator that terminates the inflammation. Although these discrepant results could be explained by the different uveitis models studied, they still raise an important concern. The exact roles of miRNAs in uveitis cannot be well elucidated by simple profiling, since the dynamic changes noted could be either the primary event that led to disease activation or the secondary physiological compensation to eliminate the inflammation.

The purpose of the present study was twofold: first, to explore the exact roles of miR-146a in the pathogenesis of EAAU, especially the relationships with NF-*κ*B, and second, to determine whether local administration of miRNAs can be a new modality among the armamentarium of uveitis management, in which the local immunotherapy possesses a steroid-sparing character and is potentially devoid of systemic adverse effects.

## 2. Materials and Methods

### 2.1. Animals

The Lewis rats (6–8 weeks old and weighing 125–160 g) were used in the experiments. All animals were treated in accordance with the recommendations in the Guide for the Care and Use of Laboratory Animals of the National Institutes of Health. The protocol was approved by the Institutional Animal Care and Use Committee of National Taiwan University College of Medicine and College of Public Health (Permit number: 20160108). All surgeries were performed under sodium pentobarbital anesthesia, and all efforts were made to minimize suffering.

### 2.2. Preparation of Antigen and Induction of EAAU

Melanin-associated antigen (MAA) was prepared according to the method proposed by Broekhuyse et al. [8, 11]. To induce EAAU, the rats were given two simultaneous injections of different MAA preparations. First, MAA was suspended in phosphate-buffered saline (PBS) and 1 : 1 emulsified in complete Freund's adjuvant (Sigma Aldrich, St. Louis, MO, USA). The suspension (0.05 mL) was injected into the left hind footpad. Second, MAA was emulsified with 1 *μ*g of purified *Bordetella pertussis* toxin (List Labs, Campbell, CA, USA) and injected into the peritoneal cavity for a total volume of 0.05 mL.

### 2.3. Animal Grouping and Treatment

The experimental animals were randomly divided into five groups and were treated accordingly. miR mimics (third-generation miRCURY LNA™, Exiqon, Vedbæk, Denmark), as referred to below, are commercially available miR molecules that simulate naturally occurring mature miRNAs.

  Group 1: rats received footpad and intraperitoneal injections of PBS as the normal control (normal group).
  Group 2: rats received footpad and intraperitoneal injections of MAA to induce EAAU (MAA group).
  Group 3: rats received MAA induction + intravitreal injection of negative miR mimic (cel-miR-39-3p, Exiqon, Vedbæk, Denmark) (MAA + negative mimic group). This group served as the sham treatment group.
  Group 4: rats received MAA induction + intravitreal injection of low-dose miR-146a (0.5 *μ*g in Lipofectamine reagent) (low-dose miR-146a group).
  Group 5: rats received MAA induction + intravitreal injection of high-dose miR-146a (1.5 *μ*g in Lipofectamine reagent) (high-dose miR-146a group).

In the current study, intravitreal injection was chosen for three reasons. First, it provides a higher intraocular concentration and avoids the systemic exposure of treatment regimen. Second, intravitreal injection is nowadays one of the most commonly performed procedures in ophthalmology, which is safe, simple, and fast. Third, the vitreous acts as a natural reservoir for drugs. Therefore, sustained release of miRNAs is possible. Weekly intravitreal injection eliminates the need for frequent dosing with topical administration or possible ocular surface discomfort following subconjunctival injection. Although intravitreal injection is not a common treatment modality for anterior uveitis in clinical practice, we think this may be a good choice to provide good intraocular concentration and reduce the suffering of the animals during this exploratory experiment. With the rats under local anesthesia with tetracaine 1%, intravitreal injections (5 *μ*L) were performed in groups 3, 4, and 5 at three time points: 0-day postimmunization (dpi) (30 min before MAA induction), 7 dpi, and 14 dpi. This selection of time points was based on the disease course of EAAU and current understanding of the stability of miRNAs. We aimed to treat the animal before the peak of disease activity, which was usually at 15 dpi. Mature miRNAs are generally considered to be much more stable than mRNAs [23]. An average miRNA half-life of 119 h (about 5 days, ranging from 101 to 225 h) has been determined by Gantier and associates. In their study, miR-146a possessed a longer half-life, slightly more than 1 week [24]. Therefore, we administered weekly injections at 0, 7, and 14 dpi. Preliminary miR-146a levels in the iris and ciliary body tissue also showed a steadily increasing trend in the miR-146a-treated groups, a result that was consistent with our treatment goal. In one diabetic rat model treated with miR-146a, weekly intravitreal injection of 1.5 *μ*g of miR-146a mimic in Lipofectamine reagent was regarded as clinically safe [25], and therefore, this dose was used in our experiment for the high-dose treatment group. To elucidate whether there was any dose-dependent treatment response, we assigned 0.5 *μ*g of miR-146a to the low-dose group.

The total numbers of animals in each group used at definite time points and the days required to perform each experiment are summarized in Table 1.

### 2.4. Clinical Examination

Daily biomicroscopic examination was performed. Disease severity was graded from 0 to 4 as follows: 0 = normal, without any anterior chamber cells or iris changes; 1 = mild iris vessel dilation and some anterior chamber cells; 2 = iris hyperemia, with some limitation in pupil dilation; 3 = miotic, hyperemic, irregular, and slightly damaged iris, with considerable flare and cells; 4 = severely damaged and hyperemic iris, with a miotic pupil filled with protein and cloudy, gel-like aqueous humor.

### 2.5. Quantification of Leukocytes in Aqueous Humor and Tissue Preparations

Aqueous humor was obtained with a 30-gauge needle (2 *μ*L), collected in silicone-treated microcentrifuge tubes (Fisher Scientific, Pittsburgh, PA, USA), and stained with 0.4% trypan blue. The numbers of leukocytes were counted under phase contrast microscopy.

After the animals were sacrificed, both eyes were harvested at each time point. The eyes were enucleated, and the iris and ciliary body were carefully isolated under an operating microscope.

### 2.6. Quantitative Measurement of Cytokine Relative mRNA Levels and Concentration with ELISA

Six cytokines of interest were chosen, for the following reasons. From our previous study, interferon- (IFN-) *γ*, IL-17, IL-12A, IL-1*β*, and IL-6 showed upregulation in the course of EAAU, while IL-10 was downregulated significantly in the course of EAAU. Therefore, mRNA levels of these six cytokines were investigated [11]. Meanwhile, IL-12 and IFN-*γ* have long been regarded as Th1-associated cytokines [26], while IL-1 and IL-17 are recognized as Th17 signature cytokines [27]. The concentration of these four key cytokines was further determined with ELISA, in order to elucidate how miR-146a might alter the disease process.

Total RNA was isolated from the iris and ciliary body with Trizol reagent (Life Technologies, Gaithersburg, MD, USA). Quantitative real-time polymerase chain reaction (PCR) was performed in triplicate. Cytokine concentration in the aqueous humor was determined with a sandwich enzyme-linked immunosorbent assay (ELISA) kit (R&D Systems, Minneapolis, MN, USA) according to the manufacturer's instructions. The ELISA was repeated three times, and the samples were diluted up to a total volume of 50 *μ*L before testing. Optical density was determined at *A*_450_ (absorbance at 450 nm) with a microplate reader (Bio-Rad, Hercules, CA, USA), and cytokine concentration was determined from standard curves using recombinant standards supplied by the manufacturer.

### 2.7. Histopathology

The enucleated eyeballs were immersed in 4% paraformaldehyde in 0.2 M phosphate buffer for 24 h. After fixation, the eyes were dehydrated with alcohol and embedded in paraffin. The specimens were then cut into 2 *μ*m sagittal sections and stained with hematoxylin and eosin to evaluate cellular infiltration in the iris and ciliary body.

### 2.8. Immunofluorescence Staining of NF-*κ*B

The tissue sections were formalin-fixed and paraffin-embedded. The slides were deparaffinized in a series of xylene solutions and rehydrated through a graded series of ethanol in PBS. Endogenous peroxidase activity was blocked by 0.3% hydrogen peroxidase in methanol, and the sections were then treated with 5% normal rat serum and incubated overnight with a monoclonal antibody against the NF-*κ*B p65 subunit (Chemicon, Temecula, CA, USA) at 4°C. Thereafter, a biotinylated secondary antibody against mouse IgG and an avidin-biotinylated peroxidase complex (Santa Cruz Biotechnology, Santa Cruz, CA, USA) were used with 3,3′-diaminobenzidine as a peroxidase substrate. All sections were counterstained with 4′,6-diamidino-2-phenylindole (DAPI).

### 2.9. Western Blotting of NF-*κ*B

The iris and ciliary body tissues were treated with radio-immunoprecipitation assay buffer and protease inhibitors to extract total protein. The extract and Laemmli buffer were mixed at a 1 : 1 ratio, and the mixture was boiled for 5 min. The samples (100 *μ*g) were then separated on 10% sodium dodecyl sulfate- (SDS-) polyacrylamide gels and transferred to polyvinylidene difluoride membranes (Millipore, Billerica, MA, USA). We incubated these membranes with anti-NF-*κ*B p65 and anti-*β*-actin antibodies. The membranes were then incubated with a horseradish peroxidase-conjugated secondary antibody and visualized by chemiluminescence (GE Healthcare, Buckinghamshire, UK). The density of the blots was quantified with image analysis software (Photoshop, Version 7.0; Adobe Systems, San Jose, CA, USA). The optical densities of each band were calculated and standardized based on the density of the *β*-actin band. All experiments were performed independently three times, and they all yielded similar results.

### 2.10. Nuclear Protein Extraction and NF-*κ*B Electrophoretic Mobility Shift Assay (EMSA)

The iris and ciliary body tissues were trypsinized, resuspended, and homogenized in buffer A [10 mM HEPES (pH 7.9), 1.5 mM KCl, 10 mM MgCl_2_, 1.0 mM dithiothreitol (DTT), and 1.0 mM phenylmethylsulfonyl fluoride (PMSF)]. The tissues were then homogenized (Dounce; Bellco Glass Co., Vineland, NJ, USA), followed by centrifugation at 5000*g* at 4°C for 10 min. The crude nuclear pellet was suspended in 200 *μ*L of buffer B [20 mM HEPES (pH 7.9), 25% glycerol, 1.5 mM MgCl_2_, 420 mM NaCl, 0.5 mM DTT, 0.2 mM ethylenediaminetetraacetic acid (EDTA), 0.5 mM PMSF, and 4 *μ*M leupeptin]. The sample was incubated on ice for 30 min and centrifuged at 12,000*g* at 4°C for 30 min. The supernatant containing the nuclear proteins was collected. A bicinchoninic acid assay kit, with bovine serum albumin (BSA) as the standard (Pierce Biotechnology, Rockford, IL, USA), was used to determine the protein concentration. EMSA was performed with an NF-*κ*B DNA-binding protein detection system (Pierce Biotechnology) according to the manufacturer's instructions. We incubated a 10 *μ*g nuclear protein aliquot with a biotin-labeled NF-*κ*B consensus oligonucleotide probe (5 ′-AGTTGAGGGGACTTTCCCAGGC-3′) for 30 min in binding buffer and then determined the specificity of the DNA/protein binding by adding a 100-fold molar excess of unlabeled NF-*κ*B oligonucleotide for competitive binding 10 min before adding the biotin-labeled probe.

### 2.11. Statistical Analysis

The results were expressed as means ± SD. Comparison of numerical data among the five groups was performed with the Kruskal-Wallis test followed by the post hoc Dunn test. A *p* value < 0.05 was considered to indicate statistical significance. All data were analyzed with Stata 12 software (StataCorp, College Station, TX, USA).

## 3. Results

### 3.1. Effect of miR-146a on Clinical Activity Score

**Figure 1:**
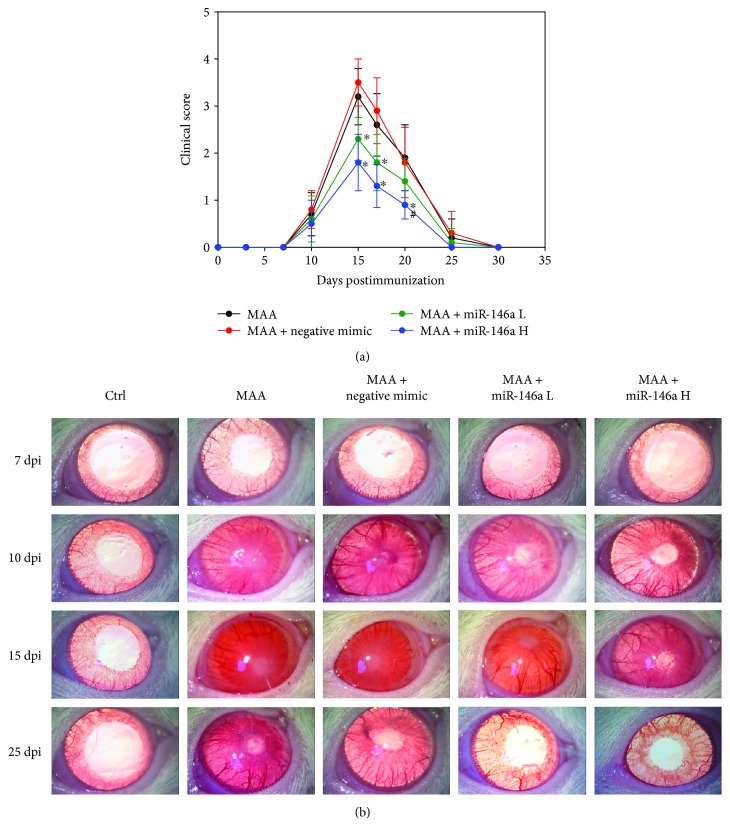
Effect of miR-146a on ocular inflammation demonstrated by clinical scores. (a) The rats in each group were examined by slit lamp biomicroscopy for clinical signs of ocular inflammation. Disease severity was clinically assessed with a scale ranging from 0 to 4. Data are expressed as means ± SD (^∗^*p* < 0.05 compared with the MAA group; ^#^*p* < 0.05 compared with the low-dose group). (b) Representative clinical photographs of the five groups at 7, 10, 15, and 25 dpi. Ctrl = control; MAA = melanin-associated antigen; miR-146a L = low-dose miR-146a; miR-146 H = high-dose miR146a.

The effect of intravitreal injection of miR-146a on the clinical activity score is shown in Figure 1. Rats injected with MAA began to develop clinical signs of EAAU at 10 dpi. The clinical score peaked at 15 dpi and declined thereafter. Rats injected with low-dose miR-146a had significantly lower clinical activity scores at 15 and 17 dpi than rats injected with MAA (*p* < 0.01 and *p* = 0.02, resp.; *n* = 10). High-dose miR-146a treatment was also associated with lower clinical activity scores at 15, 17, and 20 dpi compared with treatment with MAA (*p* < 0.01 for all paired comparisons; *n* = 10). Clinical activity scores were significantly lower in rats treated with high-dose miR-146a than in those treated with low-dose miR-146a at day 20 dpi (*p* = 0.02; *n* = 10). No significant difference in clinical activity score was noted between the MAA group and the MAA + negative mimic group over the course of the experiment.

### 3.2. Effect of miR-146a on Inflammatory Cells in the Aqueous Humor and the Iris and Ciliary Body Tissues

**Figure 2:**
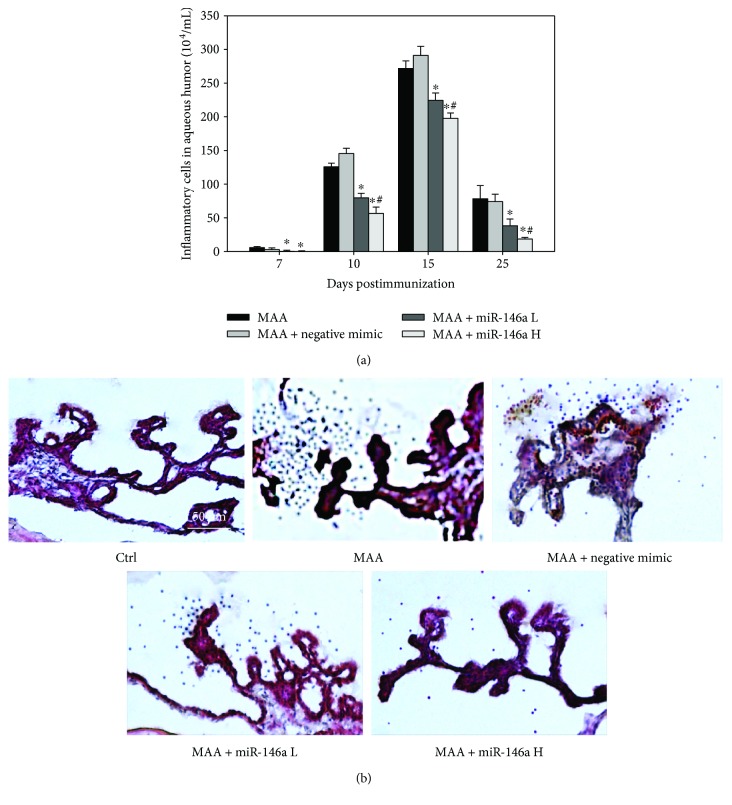
Effect of miR-146a on inflammatory cells in aqueous humor and iris and ciliary body tissues. (a) Quantification of leukocytes in the aqueous humor at 7, 10, 15, and 25 dpi. EAAU rats treated with low-dose or high-dose miR-146a had significantly lower leukocyte counts in the aqueous humor. Data are expressed as means ± SD (^∗^*p* < 0.05 compared with the MAA group; ^#^*p* < 0.05 compared with the low-dose miR-146a group). (b) Representative hematoxylin and eosin-stained sections of the iris and ciliary body of rats from the five groups. No leukocytes were seen in the control group. Profound leukocyte infiltration was noted on tissue sections from rats treated with MAA or MAA-negative mimic. The cellular response was attenuated by treatment with low-dose or high-dose miR-146a in a dose-dependent manner. Ctrl = control; MAA = melanin-associated antigen; miR-146a L = low-dose miR-146a; miR-146 H = high-dose miR146a.

The results are shown in Figure 2. The inflammatory cell count in the aqueous humor was significantly lower in rats treated with low-dose miR-146a than in rats treated with MAA at 7, 10, 15, and 25 dpi (*p* = 0.02, *p* < 0.01, *p* = 0.01, *p* = 0.04, resp.; *n* = 3). The inflammatory cell count in the aqueous humor was also significantly lower in rats treated with high-dose miR-146a than in rats treated with MAA at 7, 10, 15, and 25 dpi (*p* < 0.01 for all comparisons; *n* = 3). Compared with low-dose miR-146a-treated rats, high-dose miR-146a-treated rats had significantly lower numbers of infiltrated inflammatory cells at 10, 15, and 25 dpi (*p* = 0.04, *p* = 0.04, *p* = 0.04, resp.; *n* = 3).

The histological examination revealed that, compared with MAA treatment, treatment with low-dose or high-dose miR-146a resulted in an obvious decrease in the infiltration of leukocytes in the iris and ciliary body tissue at 14 dpi.

### 3.3. Effect of miR-146a on Expression of Cytokines

**Figure 3:**
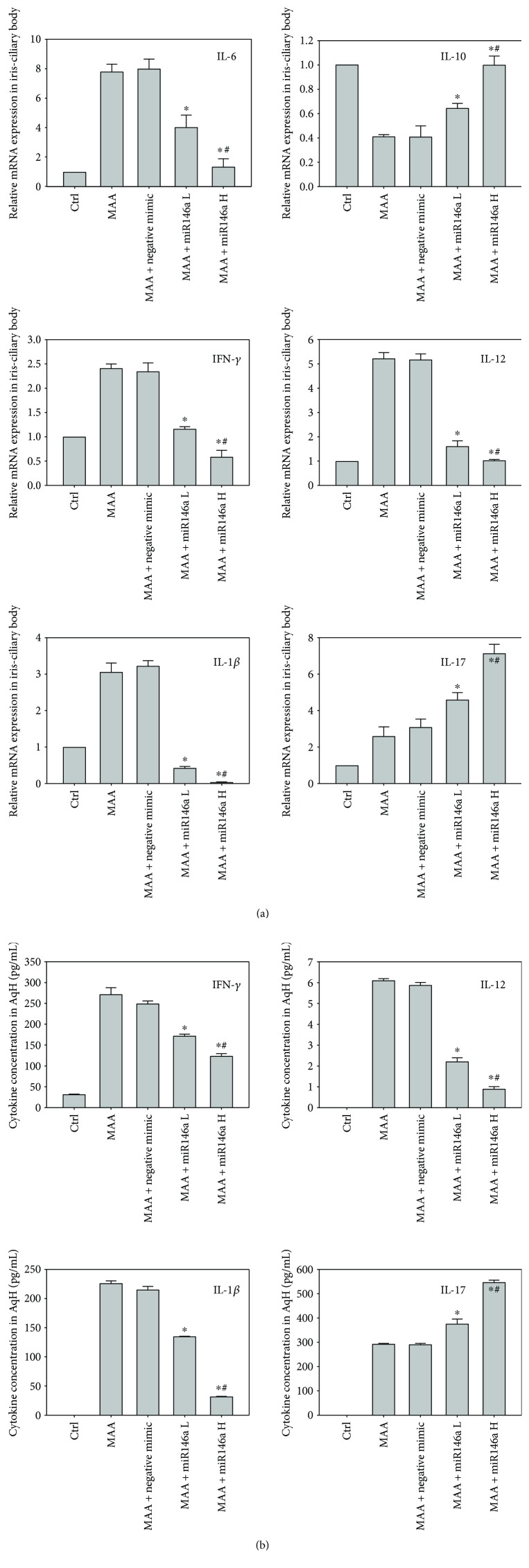
Effect of miR-146a on cytokine expression profiles. (a) Relative mRNA expression of cytokines. In comparison with the MAA group, rats treated with low-dose or high-dose miR-146a had reduced expression of IFN-*γ*, IL-12, IL-1*β*, and IL-6 mRNA and increased expression of IL-10 and IL-17. Data are expressed as means ± SD (^∗^*p* < 0.05 compared with the MAA group; ^#^*p* < 0.05 compared with the low-dose miR-146a group). (b) Aqueous concentration of cytokines by enzyme-linked immunosorbent assay (ELISA). In comparison with the MAA group, low-dose or high-dose miR-146a treatment resulted in decreased concentrations of IFN-*γ*, IL-12, and IL-1*β*, but increased concentration of IL-17. Data are expressed as means ± SD (^∗^*p* < 0.05 compared with the MAA group; ^#^*p* < 0.05 compared with the low-dose miR-146a group). Ctrl = control; MAA = melanin-associated antigen; miR-146a L = low-dose miR-146a; miR-146 H = high-dose miR146a.

A summary of the relative mRNA expression levels of cytokines in the iris and ciliary body and the cytokine concentration in the aqueous humor is shown in Figure 3. Compared with treatment with MAA, treatment with low-dose or high-dose miR-146a resulted in significant downregulation of mRNA expression of IL-6 (*p* < 0.01 in both), IFN-*γ* (*p* < 0.001 in both), IL-12 (*p* < 0.001 in both), and IL-1*β* (*p* < 0.001 in both) and upregulation of IL-10 (*p* < 0.001 in both) and IL-17 (*p* < 0.01 in both). Compared with treatment with low-dose miR-146a, treatment with high-dose miR-146a significantly reduced mRNA expression of IL-6 (*p* < 0.01), IFN-*γ* (*p* < 0.01), IL-12 (*p* = 0.01), and IL-1*β* (*p* < 0.001) and upregulation of IL-10 (*p* < 0.01) and IL-17 (*p* < 0.01) (Figure 3(a)). The aqueous concentrations of IFN-*γ*, IL-12, IL-1*β*, and IL-17 by ELISA also showed similar results (Figure 3(b)).

### 3.4. Effect of miR-146a on NF-*κ*B Expression in the Iris and Ciliary Body by Immunofluorescence

**Figure 4:**
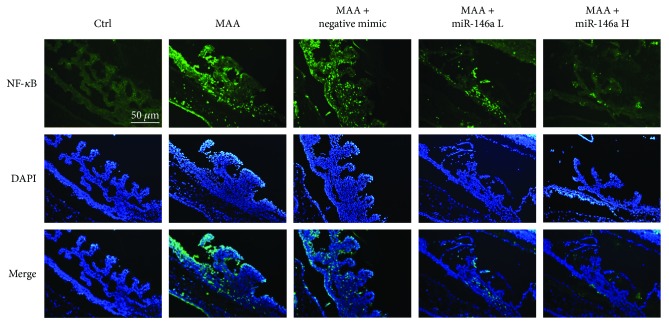
Effect of miR-146a on NF-*κ*B expression in the iris and ciliary body by immunofluorescence. NF-*κ*B expression was not observed in the control group. NF-*κ*B was profoundly expressed in the MAA and MAA + negative mimic groups. Following miR-146a treatment, reduction of NF-*κ*B expression in a dose-dependent manner was noted. The slices counterstained with DAPI were consistent with these results. Ctrl = control; MAA = melanin-associated antigen; miR-146a L = low-dose miR-146a; miR-146 H = high-dose miR146a; DAPI = 4′,6-diamidino-2-phenylindole.

The immunofluorescence results are shown in Figure 4. NF-*κ*B expression was observed in the MAA group and the MAA + negative mimic group, but not in the control group. NF-*κ*B expression was reduced following low-dose miR-146a treatment. The decrease in NF-*κ*B expression was even more profound in the group receiving high-dose miR-146a treatment. The slices counterstained with DAPI confirmed these results.

### 3.5. Effect of miR-146a on NF-*κ*B Expression in the Iris and Ciliary Body by Western Blot

**Figure 5:**
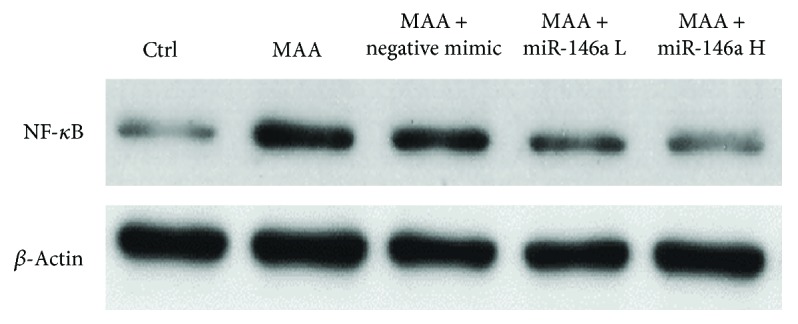
Effect of miR-146a on NF-*κ*B expression in the iris and ciliary body by Western blot. The expression of NF-*κ*B in the iris and ciliary body was obvious in the MAA group and the MAA + negative mimic group. miR-146a treatment reduced the activation of NF-*κ*B. Ctrl = control; MAA = melanin-associated antigen; miR-146a L = low-dose miR-146a; miR-146 H = high-dose miR146a.

The results are shown in Figure 5. In comparison with the control group, the expression levels of NF-*κ*B in the iris and ciliary body were significantly higher in the MAA group and the MAA + negative mimic group. Treatment with low- or high-dose miR-146a sequentially attenuated the expression of NF-*κ*B.

### 3.6. Effect of miR-146a on DNA-Binding Activity of NF-*κ*B in the Iris and Ciliary Body by EMSA

**Figure 6:**
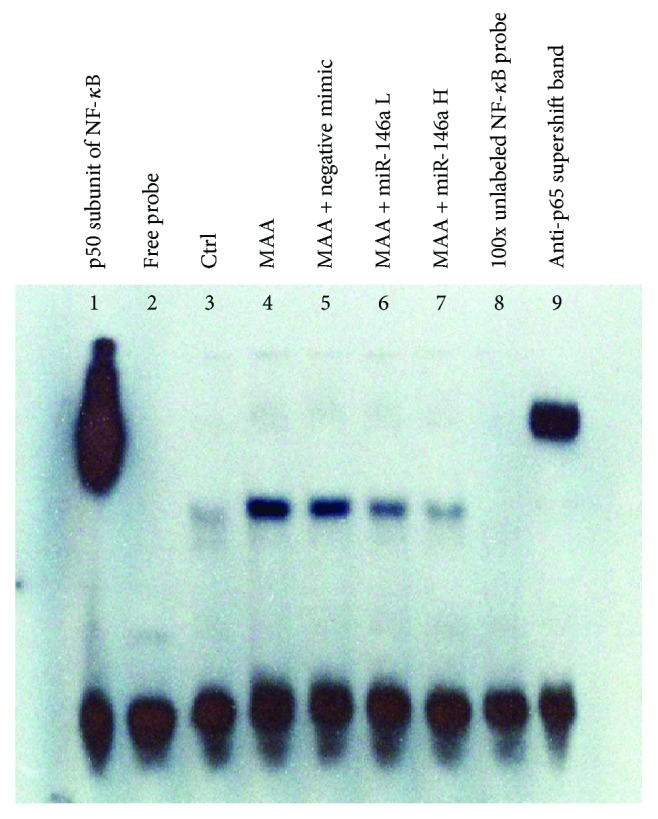
Effect of miR-146a on DNA-binding activity of NF-*κ*B by EMSA. The DNA-binding activity of NF-*κ*B was maximal in lane 4 (MAA group) and lane 5 (MAA + negative mimic group). Following treatment with miR-146a, NF-*κ*B/DNA binding activity was sequentially reduced (lanes 6 and 7). Lane 8 (100-fold unlabeled NF-*κ*B probe) confirmed these changes as NF-*κ*B specific. Ctrl = control; MAA = melanin-associated antigen; miR-146a L = low-dose miR-146a; miR-146 H = high-dose miR146a.

The results of EMSA are shown in Figure 6. The DNA-binding activity of NF-*κ*B was significantly higher in the MAA group and the MAA + negative mimic group than in the control group. In contrast, NF-*κ*B activity decreased in the miR-146a treatment groups in a dose-dependent manner.

## 4. Discussion

In our previous experiment, miR-146a and six other miRNAs showed significant dynamic changes in the course of EAAU [11]. Because of the clear relationship with NF-*κ*B, we consider miR-146a a promising candidate for the treatment of uveitis. The results of the current study have provided some valuable information. First, the effect of miR-146a-treatment in disease amelioration was well shown in clinical scores, leukocyte infiltration in the aqueous humor, and histologic findings. Second, miR-146a treatment suppressed multiple proinflammatory cytokines, such as IL-1*β*, IL-6, IL-12p35, and IFN-*γ*, although it did not suppress IL-17. Third, the results of immunofluorescence, Western blotting, and EMSA revealed that miR-146a regulated the disease process in an NF-*κ*B-dependent manner. These findings indicate a promising therapeutic potential of miR-146a in uveitis.

Generally regarded as an immunological brake, miR-146a regulates multiple pathways involving both innate and adaptive immunity. The toll-like receptor– (TLR–) NF-*κ*B pathway, which is often triggered by lipopolysaccharides from gram-negative bacteria, is one of the most important mechanisms in innate immunity. Inhibition of IRAK1, TRAF6, and ultimately NF-*κ*B is one of the pivotal actions of miR-146a [28]. This process has been noted to alter the production and effect of cytokines such as IFN-*γ*, TNF-*α*, IL-6, IL-8, and IL-1*β* [19, 29]. In our previous experiment, overexpression of IFN-*γ*, IL-17, IL-12A, IL-1*β*, and IL-6 and downregulation of IL-10 were noted in untreated EAAU [11]. Following miR-146a treatment, the expression of cytokines changed significantly, in that suppression of proinflammatory cytokines such as IL-1*β*, IL-6, IL-12p35, and IFN-*γ* and increase of anti-inflammatory cytokines such as IL-10 were noted. In comparison with its other proinflammatory counterparts, IL-17 expression was especially augmented in the miR-146a treatment group. Whether this asymmetric expansion of Th17 activity inhibits Th1 activity, reducing the clinical activity of uveitis remains unknown.

Some miRNAs show a T cell subset-specific expression pattern and hence are deeply involved in adaptive immunity [15]. An example of such a miRNA is miR-146a. miR-146a is highly expressed in regulatory T cells (Treg) [30, 31] and hence physiologically suppresses the activity of Th1 cells through STAT1 or STAT4 [32]. For instance, in people with chronic hepatitis B, miR-146a upregulation causes impaired T cell function and contributes to immune defects during chronic viral infection [32]. In addition to its expression in Treg cells, miR-146a may be relevant to Th17 lineage expression. In people with rheumatoid arthritis, it is associated with IL-17 expression in peripheral blood mononuclear cells and synovium [33].

In addition to its role in manipulating T cell lineages, current evidence also suggests the possible regulation of miR-146a in T cell receptors (TCRs). CD3^+^ (the TCR coreceptor) T cells express miR-146 in synovial tissues of people with rheumatoid arthritis [34]. T cells lacking miR-146a are hyperactive in both acute antigenic responses and chronic inflammatory autoimmune responses in one mouse model [35]. It was further noted that miR-146a dampens the TCR–NF-*κ*B pathway and inhibits AP-1 activity and IL-2 expression [35, 36]. Taken together, the evidence suggests that miR-146a is activated upon activation of TCRs and induces a negative feedback loop in the immune response through inhibition of NF-*κ*B and AP-1.

**Figure 7:**
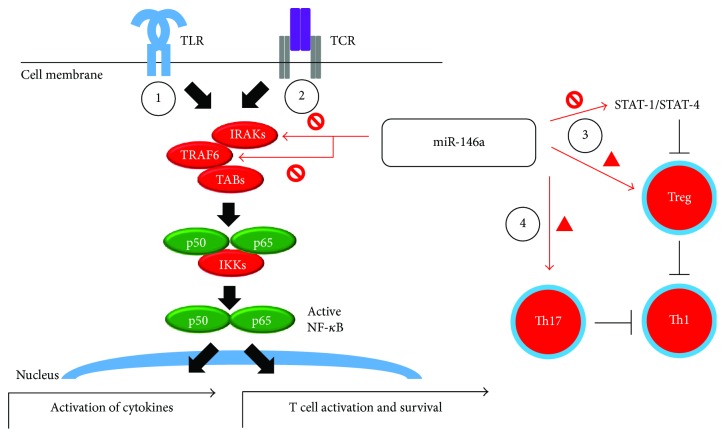
Proposed mechanisms of miR-146a in alleviating experimental autoimmune anterior uveitis (EAAU). Possible explanations of the effects of miR-146a are illustrated. (1) Blockage of the toll-like receptor–NF-*κ*B pathway, which reduces downstream secretion of proinflammatory cytokines. (2) Blockage of the TCR–NF-*κ*B pathway, which decreases T cell activation. (3) Enhancement of regulatory T cells and inhibition of Th1 axis through STAT1/4. (4) Augmentation of Th17 with dampening of Th1 lineage. TLR = toll-like receptor; TCR = T cell receptor; IRAK = interleukin-1 receptor-associated kinase; TRAF = tumor necrosis factor receptor-associated factor; IKK = I*κ*B kinase; STAT = signal transducers and activators of transcription.

In summary of the above literature, miR-146a can have multiple effects on the immune response (Figure 7). In innate immunity, blockage of the TLR–NF-*κ*B pathway, with a downstream decrease in proinflammatory cytokines, is pivotal. The activity of innate immune cells, such as macrophages, dendritic cells, and neutrophils, may be dampened following miR-146a treatment. In adaptive immunity, inhibition of the TCR–NF-*κ*B pathway is important. miR-146a possibly results in Th1 axis suppression by enhancing the activity of Treg and Th17 cells, thus halting the intraocular inflammation process in EAAU. Further analysis of T cell lineages with flow cytometry may be needed.

Expression of miR-146a has been noted to be associated with the risk of some autoimmune diseases [37]. However, studies utilizing the single-nucleotide polymorphism (SNP) approach have produced ample but inconsistent results. For example, the SNP rs2910164 G>C was found to be associated with increased risk of multiple sclerosis [38] but decreased risks of psoriasis [39] and Behcet disease [40]. Some results from human studies regarding miR-146a in intraocular inflammation were even contradictory to the current molecular knowledge that miR-146a acts as a negative autoimmunity regulator. In one human study of miR-146a and Behcet disease, individuals carrying the rs2910164 CC genotype had lower expression of miR-146a and a lower risk of developing Behcet disease [40]. In another SNP study, increased miR-146a expression was noted as a predisposing factor for pediatric uveitis [41]. The findings of both studies suggest the proinflammatory role of miR-146a. These heterogeneous results remind us the complexity of autoimmune processes in different disease models. Apart from SNP studies, our current experiment, which utilizes direct treatment with miR-146a in one uveitis model, supports the current notion that miR-146a acts as an immunological brake. These findings may also shed some light on the participation of miR-146a in systemic autoimmune diseases.

There were several limitations in this study. First, miR-146a was administered before the disease onset. Therefore, the protective effect against EAAU attack could not completely imply the therapeutic effect following the emergence of uveitis. Second, although intravitreal injection achieved a stable and predictable intraocular concentration and is performed frequently in clinical practice, it was still considered relatively invasive. If possible, topical or subconjunctival miR-146a administration would be a more realistic treatment choice in the future. Third, although the alterations of either Th1/Th17 signature cytokines were significantly noted, flow cytometry was not performed to investigate the real T cell status. Nevertheless, the current study is unique in its in vivo design, which helps establish the real influence of miR-146a in a uveitis model by a direct therapeutic approach. Meanwhile, the profiles of clinical inflammation, aqueous cell infiltration, histology, NF-*κ*B expression, and cytokine alterations following miR-146a treatment may be a useful guide for further research.

In conclusion, the current study demonstrated the effects of intravitreal injection of miR-146a in alleviating EAAU. miR-146a dampened intraocular inflammation through the inhibition of NF-*κ*B, resulting in reduced leukocyte infiltration and reduced production of proinflammatory cytokines. With the advantages of the possibility of local treatment and its steroid-sparing effect, miR-146a may be a promising choice for the treatment of uveitis.

## Figures and Tables

**Table 1:** Summary of total number of animals at each time point in each group per experiment and days to perform the experiments.

Experiments	Number of animals at each time point in each group	Days postimmunization (*n*)
Clinical scores	10	Day 0, 7, 10, 15, 17, 20, 25
Leukocyte counts	3	Day 0, 7, 10, 15, 25
Cytokine mRNA and ELISA	3	Day 15
Histology	3	Day 15
Western blot analysis	3	Day 15
Immunofluorescence of NF-*κ*B	3	Day 15
EMSA	3	Day 15
